# Metastatic Melanoma Cells Evade Immune Detection by Silencing STAT1

**DOI:** 10.3390/ijms16024343

**Published:** 2015-02-17

**Authors:** JoDi Lynn Osborn, Susanna F. Greer

**Affiliations:** Division of Cellular Biology and Immunology, Department of Biology, Georgia State University, Atlanta, GA 30303, USA; E-Mail: josborn1@gsu.edu

**Keywords:** MHC II, melanoma, STAT1, immunosurveillance

## Abstract

Transcriptional activation of major histocompatibility complex (MHC) I and II molecules by the cytokine, interferon γ (IFN-γ), is a key step in cell-mediated immunity against pathogens and tumors. Recent evidence suggests that suppression of MHC I and II expression on multiple tumor types plays important roles in tumor immunoevasion. One such tumor is malignant melanoma, a leading cause of skin cancer-related deaths. Despite growing awareness of MHC expression defects, the molecular mechanisms by which melanoma cells suppress MHC and escape from immune-mediated elimination remain unknown. Here, we analyze the dysregulation of the Janus kinase (JAK)/STAT pathway and its role in the suppression of MHC II in melanoma cell lines at the radial growth phase (RGP), the vertical growth phase (VGP) and the metastatic phase (MET). While RGP and VGP cells both express MHC II, MET cells lack not only MHC II, but also the critical transcription factors, interferon response factor (IRF) 1 and its upstream activator, signal transducer and activator of transcription 1 (STAT1). Suppression of STAT1 *in vitro* was also observed in patient tumor samples, suggesting STAT1 silencing as a global mechanism of MHC II suppression and immunoevasion.

## 1. Introduction

**Figure 1:**
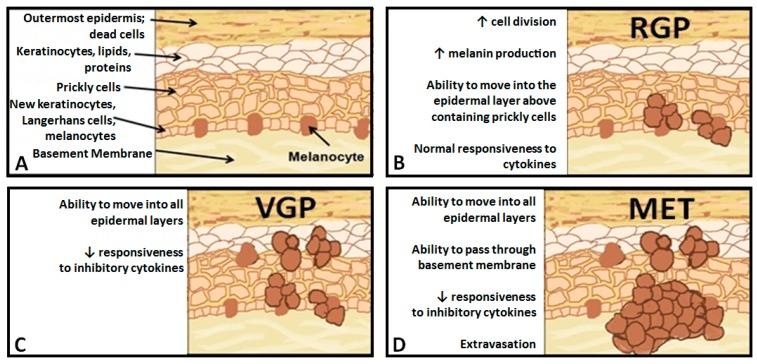
Representation of the progression from healthy melanocyte, to the radial growth phase (RGP), to the vertical growth phase (VGP), to metastatic (MET) melanoma. (**A**) Healthy melanocytes exist in a fixed ratio to keratinocytes in the basal layer of the epidermis; (**B**) RGP cells exhibit uncontrolled cell division and over-production of melanin; (**C**) VGP cells can spread throughout the epidermis and no longer respond to proliferation, inhibiting cytokines; and (**D**) MET cells are able to break through the basement membrane and enter the circulation.

Skin cancer is the most common form of cancer diagnosed in the United States. Despite comprising only 5% of skin cancers, malignant melanoma is the leading cause of skin cancer-related deaths annually. Melanoma progresses through stages, from the radial growth phase (RGP) to the vertical growth phase (VGP) to metastatic, with distinct morphologic phenotypes; However, the molecular changes associated with these transitions are not well defined [1]. Healthy melanocytes exist in a fixed ratio to keratinocytes in a heterogeneous environment that includes fibroblasts, endothelial cells and resident immune effector cells (Figure 1A) [2]. Initial gene dysregulation leads to the development of dysplastic nevi, which exhibit phenotypes, including, but not limited to, over-production of melanin [3]. RGP melanomas are characterized by uncontrolled cellular division, as well as the ability to spread within the epidermis, but generally resemble healthy melanocytes at the molecular level (Figure 1B) [4,5]. VGP melanomas are capable of spreading throughout skin layers and develop resistance to paracrine growth inhibition via cytokines secreted by surrounding endothelial cells (Figure 1C) [6,7]. Finally, metastatic (MET) melanomas develop the ability to intravasate, allowing them to establish secondary tumors elsewhere in the body (Figure 1D) [8]. While the mechanisms underlying the transition between RGP, VGP and MET are not fully understood, the genes involved in the transition and their contributions to the deadly nature of metastatic melanoma are beginning to be elucidated.

The immune system is able to specifically detect and target neoplastic cells through the process of immunosurveillance [9]. The promise of cancer immunotherapy is to use immunosurveillance to eliminate cancer cells without harming normal tissues, thus with fewer side effects. Immunotherapy approaches have been tested against malignant melanoma, and while detectable outcomes have been induced, the clinical results have largely been disappointing [10]. Escape from tumor immunosurveillance is a major mechanism leading to the lethality of metastatic melanomas [11]. A first step in immunosurveillance is the recognition of tumor peptide antigens by T-cells of the immune system. Tumor antigens are presented by cell-surface glycoproteins, termed major histocompatibility complex (MHC) molecules I and II [12]. MHC I molecules present intracellular peptides and are expressed on nucleated cells, whereas MHC II molecules present extracellular peptides constitutively on professional antigen presenting cells and inducibly on nucleated cells [12].

In the case of tumors, MHC I molecules present tumor-associated antigens (TAA) to CD8^+^ cytotoxic T-cells, and MHC II molecules present TAAs to CD4^+^ helper T-cells, thus facilitating an anti-tumor immune response [13]. In these contexts, T-cells recognize dysplastic cells as “altered self” and eradicate the tumor [9]. Traditionally, CD8^+^ T-cells have been considered the major mediators of effective anti-tumor immune responses, and suppression of MHC I in tumors is well studied. Decreases in MHC I expression, and, thus, antigen presentation to CD8^+^ T-cells, negatively impacts tumor prognosis in numerous cancer types [14]. The observation that antigen presentation via MHC I is critical in tumors is supported by exomic sequencing studies identifying that presentation of mutated TAAs on MHC I led to increased tumor burden in mice [15]. However, a growing number of studies indicate limited anti-tumor activity of CD8^+^ T-cells alone [16,17,18,19]. The helper function of anti-tumor CD4^+^ T-cells improves the efficacy of anti-tumor CD8^+^ T-cells; seen early on in studies where transfecting tumor cells with MHC class II genes resulted in increased anti-tumor immune responses [20,21]. Multiple studies now indicate that CD4^+^ T-cells enhance the anti-tumor response of CD8^+^ T-cells [22,23,24,25,26,27]. Indeed, CD4^+^ T-cells can also eliminate tumor cells in the absence of CD8^+^ T-cells [28,29,30,31]. Collectively, these findings suggest that CD4^+^ T-cells are powerful antitumor effector cells.

CD4^+^ T-cells recognize peptides bound to the groove of MHC II molecules [32]. As MHC II cell surface expression can be induced in nucleated cells by the cytokine, interferon gamma (IFN-γ), through the JAK/STAT signaling cascade [33], the molecular mechanisms regulating the MHC II status of tumor cells is of clear importance. It has been well established that IFN-γ plays substantial roles in both anti-viral and pro-immune responses [34]. Both MHC I and MHC II are IFN-γ inducible, further emphasizing the significance of this type II interferon in immunosurveillance. In the context of MHC II transcription, IFN-γ binds to IFN-γ receptor 1 (IFN-γ R1), leading to dimerization of IFN-γ R1 and IFN-γ R2 [35]. The cytoplasmic tails of the hetero-dimeric IFN-γ Receptors are cross-phosphorylated, allowing binding of Janus kinase (JAK) 1 and JAK2 [36]. JAK1 and JAK2 are subsequently phosphorylated, leading to recruitment and binding of signal transducer and activator of transcription 1 (STAT1) [37]. Phosphorylated STAT1 then forms a homodimer known as γ-activated factor (GAF) [38]. GAF next translocates to the nucleus, where it binds the γ-activation sequence (GAS) on the promoters of interferon response element (IRF) 1 and IRF2 [39]. An IRF1 and IRF2 heterodimer along with GAF binds promoter IV of the class II transactivator (CIITA). CIITA is necessary, but not sufficient, for the transcription of MHC II and lacks intrinsic DNA binding capabilities [40]. MHC II transcription requires the presence of the enhanceosome complex to which CIITA binds [40]. The enhanceosome is comprised of regulatory factor X (RFX), nuclear factor Y (NFY) and cAMP regulatory element binding protein (CREB) [41]. CIITA binding at the enhanceosome allows binding of RNA polymerase II, leading to transcription of MHC II [42]. It has previously been reported that metastatic melanoma lacks cell surface expression of MHC II, which leads to tumor escape from immunosurveillance [43]. We therefore sought to determine the mechanisms underlying the silencing of MHC II in metastatic melanoma.

## 2. Results

### 2.1. MHC II Is Increasingly Suppressed in RGP, VGP and MET Cells

**Figure 2:**
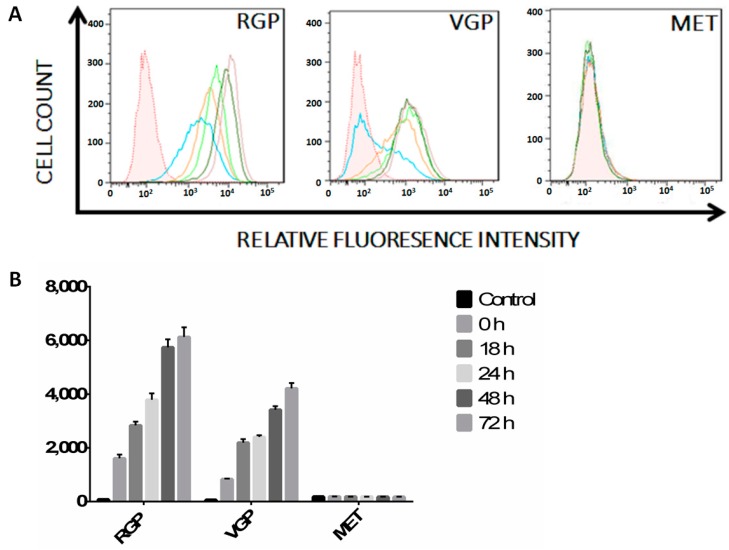
Flow cytometric analysis of MHC II cell surface expression in RGP, VGP and MET cells. (**A**) Cells were treated with IFN-γ for 0 h (blue), 18 h (orange), 24 h (lime green), 48 h (dark green) or 72 h (mauve). Unstained cells (shaded histogram) are shown as a control. Live cells were stained with APC-conjugated antibody against MHC II, were fixed in 2% paraformaldehyde and were then analyzed on a Fortessa flow cytometer. RGP cells demonstrate basal expression of MHC II that increases following IFN-γ stimulation. VGP cells demonstrate less basal expression of MHC II, with an increase upon IFN-γ stimulation comparable to the basal expression seen in radial growth phase (RGP) cells. MET cells lack cell surface expression of MHC II with, or without, stimulation with IFN-γ. The data shown are representative of a minimum of three experimental replicates; (**B**) Mean fluorescence intensity of the histograms seen in (**A**) normalized to the isotype control. Data are the average of three experimental replicates.

Despite the ability of the immune system to detect and eradicate tumor cells, tumor immunosurveillance often fails [44]. Selective pressure leads to tumor cells evolving to downregulate immunomodulatory molecules, a phenomenon known as tumor immunoediting [45]. While metastatic melanoma cells often lack MHC II surface expression, the mechanisms supporting MHC II suppression by metastatic melanoma remain unknown [43]. We used flow cytometry to determine the expression levels of MHC II during the progression of melanoma. Radial growth phase (RGP) (Figure 2) cells express basal levels (blue) of MHC II as compared to unstained control (shaded), with MHC II increasing upon stimulation. Relative fluorescence intensity (*x*-axis) representing APC-MHC II complexes increases after 18 h (orange) and continues to increase throughout 24 h (light green), 48 h (dark green) and 72 h (mauve) of IFN-γ stimulation. In comparison, vertical growth phase (VGP) (Figure 2) cells express lower basal MHC II, with a majority of cells being APC-MHC II-negative in the absence of IFN-γ stimulation (blue). Similar to RGP cells, VGP cells demonstrate increased MHC II cell surface expression throughout 72 h of stimulation; albeit to a lesser extent. In contrast with both RGP and VGP, metastatic (MET) (Figure 2) cells lack constitutive or inducible cell surface expression of MHC II. These data imply that MHC II cell surface expression decreases as melanoma cells progress through the RGP and VGP stages and confirm previously reported findings of MET cells lacking cell surface MHC II [43].

### 2.2. Melanoma Cells Remain IFN-γ Responsive throughout Disease Progression

**Figure 3:**
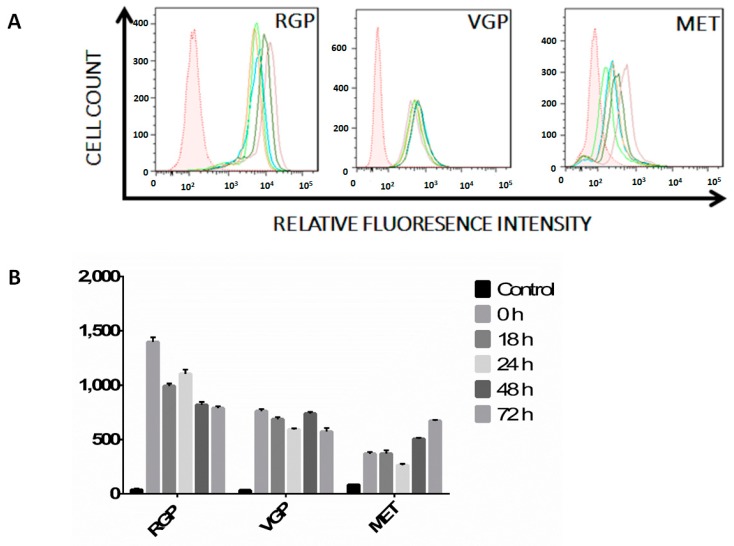
(**A**) Flow cytometric analysis of IFN-γ receptor expression. Cells were treated with IFN-γ for 0 h (blue), 18 h (orange), 24 h (lime green), 48 h (dark green) or 72 h (mauve). Unstained cells (shaded histogram) are shown as a control. Live cells were stained with PE-conjugated antibody against CD119 (IFN-γ R1), were fixed in 2% paraformaldehyde and were then analyzed on a Fortessa flow cytometer. RGP cells express high levels of the IFN-γ receptor as compared to the unstained control. VGP cells express low levels of the IFN-γ receptor with no change throughout 72 h of stimulation. MET cells express varying amounts of IFN-γ receptor throughout 72 h of stimulation. Changes in receptor expression between time points is not statistically significant (*p* > 0.05). The data shown are representative of a minimum of three experimental replicates; (**B**) Mean fluorescence intensity of histograms seen in (**A**) normalized to the isotype control. The data shown are the average of three experimental replicates.

As cell surface expression of MHC II on nucleated cells requires stimulation with the pro-inflammatory cytokine, IFN-γ, we questioned whether the MET cell line was capable of responding to stimulation [46]. We performed flow cytometry experiments to determine the expression levels of this cell surface receptor, focusing on IFN-γ R1, because it is the heterodimer constituent to which IFN-γ binds prior to heterodimerization [35]. Furthermore, mutations in IFN-γ R2 slightly decrease cell surface expression attenuation of signaling, but do not impart a total loss of IFN-γ responsiveness [47,48]. As expected, RGP cells express high levels of cell surface IFN-γ R1 with no significant change in receptor expression during cytokine stimulation (Figure 3—RGP). VGP cells express moderate levels of IFN-γ R and also show no change during stimulation (Figure 3—VGP). MET cells express moderate to low levels of IFN-γ R1 (Figure 3—MET), where expression levels varied throughout stimulation, but not to a significant extent. Together, these data indicate that RGP, VGP and MET cells are each capable of responding to interferon stimulus.

### 2.3. MET Cells Express Both Janus Kinase 1 (JAK1) and JAK2

**Figure 4:**
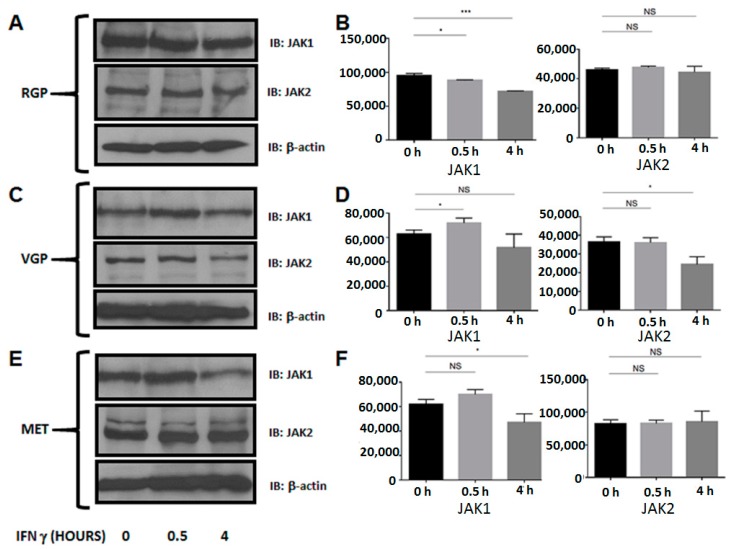
Western blot analysis of JAK1 and JAK2 expression. Cells were stimulated with IFN-γ for 0, 0.5 or 4 h. Lysates were cleared of cellular debris, and equal concentrations of protein were separated via SDS-PAGE. Proteins were identified by incubating nitrocellulose with antibodies against JAK1 (RGP, VGP, MET; **top**) or JAK2 (RGP, VGP, MET; **middle**). β-Actin was used as a loading control (RGP, VGP, MET; **bottom**). (**A**–**F**) JAK1 and JAK2 are constitutively expressed in RGP, VGP and MET cells. The data shown are representative of a minimum of three experimental replicates. The *y*-axis represents Pixel Total (quantification of A, C, E). NS: Not Significant; * *p* < 0.05, *** *p* < 0.001

MHC II transcription is a product of the Janus kinase (JAK) signaling cascade [36]. Upon IFN-γ binding to the IFN-γ receptor on the cell surface, JAK1 and JAK2 bind the cross-linked receptor and cross-phosphorylate one another, leading to STAT1 activation [37]. The fact that both JAK1 and JAK2 are imperative in the signaling cascade required for MHC II cell surface expression is well established, and their abrogation leading to decreased MHC II has been seen in certain bacterial infections [49]. Therefore, we performed Western blots to determine the expression levels of JAK1 and JAK2 during the course of melanoma development. We observed that in all three cell lines, JAK1 and JAK2 are expressed in the presence or absence of IFN-γ stimulation (Figure 4). These data show that JAK1 and JAK2 are intact in metastatic melanoma and are not the underlying cause of MHC II silencing in these cells.

### 2.4. Metastatic Melanoma Cells Lack the Interferon Response Factor, IRF-1

**Figure 5:**
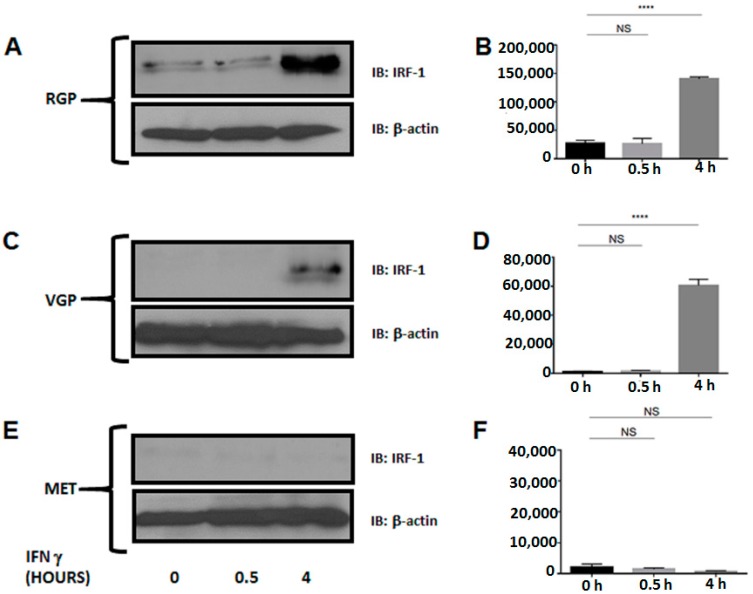
Western blot analysis of IRF-1 expression. Cells were stimulated with IFN-γ for 0, 0.5 or 4 h. Lysates were cleared of cellular debris, and equal concentrations of protein were separated via SDS-PAGE. Proteins were identified by incubating nitrocellulose with antibodies against IRF-1 (RGP, VGP, MET; **top**). β-Actin was used as a loading control (RGP, VGP, MET; **bottom**). (**A**,**B**) IRF-1 is expressed in RGP cells following four hours of IFN-γ stimulation; (**C**,**D**) In VGP cells, IRF-1 is expressed to a greater extent after four hours of stimulation, compared to RGP; (**E**,**F**) MET cells lack IRF-1 expression following 4 h of IFN-γ stimulation. *y*-axes (**B**,**D**,**F**) represent Total Pixels (Quantification of **A**,**C**,**E**). NS: Not Significant; **** *p* < 0.0001.

Downstream from JAK1 and JAK2 and following IFN-γ stimulation, IRF-1 forms a heterodimer with IRF-2 and binds CIITA PIV, leading to transcription of the class II transactivator [50]. Because IRF-1 is necessary for CIITA transcription, we investigated the expression of IRF-1 with and without interferon stimulation (Figure 5). We and others have determined that IRF-1 is expressed at its maximum level after 4 h of stimulation in near normal cells [51,52]. As expected, IRF-1 is expressed following four hours of IFN-γ stimulation in RGP and VGP cells. However, MET cells lack IRF-1 expression despite interferon stimulation. These data imply that silencing of MHC II in metastatic melanoma is due to silencing of IRF-1.

**Figure 6:**
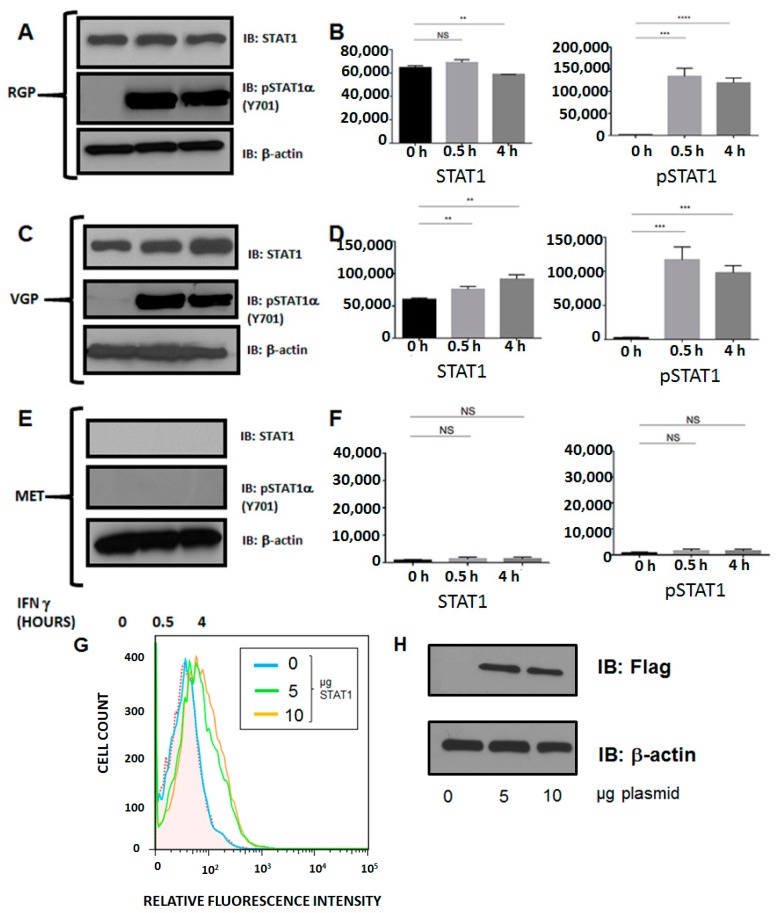
Western blot analysis of STAT1 expression and phosphorylation. Cells were stimulated with IFN-γ for 0, 0.5 or 4 h. Lysates were cleared of cellular debris, and equal concentrations of protein were separated via SDS-PAGE. Proteins were identified by incubating nitrocellulose with antibodies against STAT1 (RGP, VGP, MET; top) or pSTAT1 (RGP, VGP, MET; middle). β-Actin was used as a loading control (RGP, VGP, MET; bottom). (**A**,**B**) STAT1 is constitutively expressed and phosphorylated at tyrosine 701 (Y701) following 30 minutes of IFN-γ stimulation in RGP cells; (**C**,**D**) STAT1 expression and phosphorylation in VGP cells is similar to RGP; (**E**,**F**) STAT1 expression is absent in MET cells. The data shown are representative of at least three experimental replicates. *y*-axes (**B**,**D**,**F**) represent Total Pixels (Quantification of **A**,**C**,**E**). NS: Not Significant; (**G**) MET cells + STAT1. MET cells were transfected with 0, 5, 10 µg of Flag-STAT1 for 24 h followed by 48 h of IFN-γ stimulation. Cell surface expression of MHC II was analyzed via flow cytometry; and (**H**) Expression of Flag-STAT1 following 24 h of incubation with transfection reagent:plasmid complexes. Both 5 µg (light green) and 10 µg (orange) of STAT1 led to restoration of MHC II on the cell surface, compared to non-transfected cells (light blue) and the unstained control (shaded). ** *p* < 0.005, *** *p* < 0.001, **** *p* < 0.0001.

**Figure 7:**
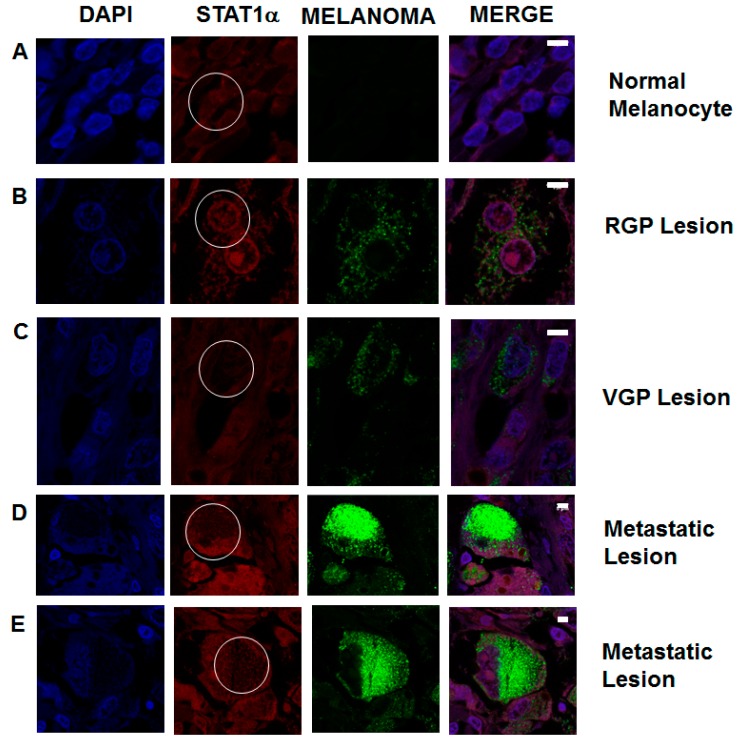
Immunofluorescence staining of STAT1 expression in non-metastatic and metastatic melanocytic lesions from patient samples. Tissue microarrays of patient samples were de-waxed, rehydrated and stained for the expression of STAT1 (red) and metastatic melanoma biomarkers (green). Cell nuclei are stained with DAPI (blue). Regions of interest are circled in white on the STAT1 panels. (**A**) Normal melanocytes show constitutive STAT1 expression; (**B**) Primary tumors characterized as RGP show a slight decrease in STAT1 expression; (**C**) VGP melanoma lesions show an increase in melanoma biomarkers, as well as a decrease in STAT1; (**D**,**E**) Metastatic melanoma lesions show a marked increase in metastatic melanoma biomarkers, which correlates with a decrease in STAT1. These data show a correlation between biomarkers of metastasis and the decrease in STAT1 expression in patient tumor samples. Data are representative of images taken from approximately 150 patient samples. Scale bars represent 5 µm.

### 2.5. Silencing of MHC II in Metastatic Melanoma Is the Result of Dysregulation of Basal STAT1α Expression

IRF-1 is required for CIITA and, thus, MHC II expression following stimulation with the pro-inflammatory cytokine, IFN-γ. The γ-activated sequence (comprised of a homodimer of STAT1) is required for both IRF-1 and CIITA expression. We hypothesized that dysregulated STAT1 activation was the cause of the silencing of both IRF-1 and CIITA, leading to the lack of MHC II cell surface expression. Western blot analysis verified that STAT1 is constitutively expressed and is inducibly phosphorylated upon interferon stimulation in RGP and VGP cells. In contrast, MET cells lack not only phosphorylated STAT1, but also lack constitutive STAT1 protein expression. Conversely, re-introduction of STAT1 into MET cells restores cell surface expression of MHC II (Figure 6).

### 2.6. STAT1 Expression Is Decreased in Patient Metastatic Melanoma Samples

To investigate if the observations seen *in vitro* mirror the metastatic melanoma samples, we examined STAT1 expression levels in patient tumor samples. In normal melanocytes from a benign nevus, STAT1 is expressed throughout the cell (Figure 7A). RGP lesions show a visual decrease in STAT1 expression. Melanoma cells are identified by upregulation of biomarkers, as seen in green. (Figure 7B). VGP lesions show a further decrease in STAT1 expression, which correlates with an increase in melanoma biomarker expression (Figure 7C). Metastatic lesions from lymph nodes (Figure 7D) and bone (Figure 7E) both show a marked decrease in STAT1 expression. Data are representative of over 150 samples. These data show that in clinical samples, STAT1 expression has an inverse correlation with upregulation of biomarkers for metastatic melanoma.

## 3. Discussion

Tumor immunosurveillance refers to the ability of the immune system to detect and, ideally, respond to neoplastic cells. Successful immunosurveillance depends on the presentation of TAAs to CD8^+^ cytotoxic T-cells and to CD4^+^ T-helper cells via MHC I and MHC II, respectively. The suppression of either one or both of these major histocompatibility complexes is a common mechanism by which tumor cells avoid immunosurveillance. Numerous silencing mechanisms have been elucidated, including epigenetic silencing and genomic deletion of key mediators involved in MHC cell surface expression [53,54,55,56].

The goal of this study was to determine the mechanisms underlying previous observations of silencing of IFN-γ-inducible MHC II expression in metastatic melanoma. We report here that MHC II is suppressed as melanoma cell lines evolve from RGP to VGP to MET. We further determined that despite gradual suppression of MHC II, each melanoma cell line remained IFN-γ responsive throughout simulated disease progression. Continued cell surface expression of the IFN-γ receptor led us to inspect the remaining components of the IFN signaling cascade leading to MHC II cell surface expression. Following IFN-γ stimulation, JAK1 and JAK2 bind to the intercellular domains of the IFN-γ receptor [36]. RGP, VGP and MET cell lines all express both JAK1 and JAK2, further indicating that the major components of the MHC II signaling pathway are intact, regardless of the stage of melanoma progression. Upon further investigation of the JAK/STAT pathway, we found that metastatic melanoma cells lack the interferon response factor IRF-1. IRF-1 requires activated STAT1 (phosphorylated at Y701 to form a homodimer) for transcriptional activation. MET cells not only lack phosphorylated STAT1, but lack basal expression of unphosphorylated STAT1, as well. From these results, we conclude that the suppression of MHC II on the cell surface of these MET melanoma cell lines is due to silencing of STAT1. To determine if a similar phenomenon is present in patient samples, we compared STAT1 expression levels in metastatic and non-metastatic melanocytic lesions. Within primary tumors and secondary metastases, we see significant decreases in STAT1 expression as cells gain metastatic ability. We are currently investigating the molecular mechanisms by which STAT1 is suppressed in metastatic melanoma.

Studies over the past decade have led to the discovery of varying causal mechanisms of melanoma immune escape centered on the suppression of antigen-presenting molecules. The majority of these studies have focused on MHC I, due to its importance in activation of cytotoxic T-cells. Rodriguez and colleagues investigated cell lines from the European Searchable Tumor Line Database (ESTDAB) and found multiple metastatic melanoma cell lines that suppress MHC I with differences in the mechanisms of MHC I suppression [53]. The melanoma cell line ESTDAB-004 expresses STAT1, but the STAT1 expressed lacks phosphorylation at Y701. In contrast, lack of MHC I in ESTDAB-159 (GR-mel-3) cells was due to promoter methylation of IRF-1. Methylation was also found to be the cause of MHC I silencing in MSR3-mel cells, but in this case, hypermethylation occurred at the MHC I promoter itself [54]. Additionally, in metastatic melanoma Colo857 cells, MHC is absent due to a genomic deletion of JAK2 [55].

Silencing of MHC I molecules has been observed at nearly every level of the JAK/STAT signaling cascade. Because STAT1 is involved in both MHC I and MHC II expression, it is likely that the observed effects of STAT1 silencing in MHC I impairment are mirrored in the impairment of MHC II. Few reports are available that have investigated the silencing of MHC II in metastatic melanoma. One early study shows that MHC II silencing in the ocular melanoma cell line, Mel202, is the result of epigenetic silencing of CIITA [56]. In this study, ectopic expression of CIITA restored MHC II cell surface expression. Aside from the report indicating that the metastatic melanoma cell line 1205lu does not express cell-surface MHC II [43], little has been reported in regards to the expression of MHC II in skin melanomas. In concurrent studies with our own, it was recently shown that STAT1 is silenced in two additional metastatic melanoma cell lines, SK-Mel-28 and MM96 [57]. The RE-1 silencing transcription (REST) complex was shown to regulate STAT1 in this study. REST is responsible for silencing neuronal genes in non-neuronal cells, but has also been shown to play a role in some genetic disorders [58]. Interestingly, STAT1 contains an RE1 binding site, and it was shown that this is the mechanism by which REST silences STAT1. These findings revealed a previously undocumented mechanism of downregulation of STAT1 in melanoma. The above observations underscore the variation in underlying causes of MHC silencing in metastatic melanoma. In contrast to the Wistar melanoma cell lines utilized in our study, the aforementioned cell lines are not categorized by progression status. To our knowledge, our study is the first to compare MHC suppression between RGP, VGP and MET melanoma cell lines.

Silencing of STAT1 may be one mechanism by which melanoma can evade immune detection and, thus, increase its metastatic potential. By investigating STAT1 expression in patient tumors, oncologists may be able to employ targeted immunotherapies, thus increasing a patient’s immune response to metastatic melanoma. The ability to introduce plasmid DNA (pDNA) into human subjects has become an increasingly common part of clinical trials. STAT1-encoding pDNA can be introduced into tumors via electroporation [59,60]. Additionally, techniques are in development to use mRNA in lieu of pDNA to augment gene expression in tumors [61]. By developing mechanisms to introduce STAT1 DNA into tumors lacking the protein and by treating the patient with a course of interferon, it may be possible to induce MHC I and MHC II expression on the cell surface of melanoma, leading to an increased response by the patient’s own immune system. Immunotherapy has gained notoriety in recent years, because it is less toxic to patients than chemotherapy and irradiation. The timing of immunotherapeutic intervention has been shown to drastically affect patient outcomes. Observations of the importance of timely intervention with immunotherapy have led to the establishment of the Copenhagen Prospective Personalized Oncology (CoPPO) program in which samples from newly diagnosed patients undergo multiple tests, including expression arrays, RNA sequencing and SNP analysis, to determine the expression levels of therapeutic targets. Knowing the mutations leading to neoplasms will allow clinicians to tailor treatment and, ideally, improve patient prognosis [62]. Consequently, determining the multitude of underlying causes of tumors is imperative to improving personalized medicine.

## 4. Experimental Section

### 4.1. Cell Lines

Wistar melanoma (WM) cell lines representing the radial growth phase (WM-35), vertical growth phase (WM-1366) and metastasis (1205Lu) were purchased from the Coriell Cell Repositories (Coriell Institute for Medical Research, Camden, NJ, USA). Cell lines were maintained in 4:1 MDB153:Liebowitz L-15 Media (Sigma-Aldrich, St. Louis, MO, USA) and 2% FBS (Atlas Biologicals, Fort Collins, CO, USA). Cells were cultured at 37 °C in 5% CO_2_.

### 4.2. Western Blots

Cells were treated with 50 U/mL IFN-γ (Peprotech, Rocky Hill, NJ, USA) for 0 hours, 0.5 h or 4 h. Post-stimulation, cells were lysed in NP-40 lysis buffer (20 mM Tris pH 8.0, 0.14 M KCl, 10% Nonadet P-40, 5 mM EDTA, 20 mM NaCl, 1 mM DTT) supplemented with eComplete Protease Inhibitors (Roche Applied Science, Indianapolis, IN, USA). Clarified lysates were normalized for protein concentration, separated via SDS-PAGE, transferred to nitrocellulose and blotted for the protein of interest with specific antibodies as follows: IRF-1, STAT-1 (Santa Cruz Biotechnology, Santa Cruz, CA, USA), phospho-STAT1 (Y701) (BD Biosciences, San Jose, CA, USA) and JAK1, JAK2 (Cell Signaling Technology, Boston, MA, USA). Blots were incubated with appropriate secondary antibodies conjugated to HRP and developed with Hyglo™ (Denville Scientific, South Plainfield, NJ, USA), per the manufacturer’s instructions. HRP conjugated β-actin antibodies (Cell Signaling Technology, Boston, MA, USA) were used as loading controls.

### 4.3. Flow Cytometry

Cells were treated for 0, 18, 24, 48 or 72 h with IFN-γ at a concentration of 50 U/mL. Post-stimulation, cells were harvested with Accutase (EMD Millipore, Billerica, MA, USA). After washing with PBS, cells were stained with fluorophore-conjugated-specific antibodies as follows: PE-CD119 (IFN-γ R1) or APC-HLADR (MHC II) (Biolegend, San Diego, CA, USA). Cells were then fixed in 2% paraformaldehyde and analyzed on an LSRFortessa Flow Cytometer (BD Biosciences, San Jose, CA, USA). Data were analyzed using FlowJo Software (Tree Star, Ashland, OR, USA).

### 4.4. Immunofluorescence

Formalin-fixed paraffin embedded (FFPE) tissue microarrays (Biomax, Rockville, MD, USA) were dewaxed in two 20-min washes with xylenes. Microarrays were rehydrated and blocked as previously described [63]. Slides were incubated with primary antibodies for STAT1 (Santa Cruz Biotechnology, Santa Cruz, CA, USA) or the metastatic melanoma biomarkers, HMB45 and MART-1 (Abcam, Cambridge, MA, USA), for one hour at room temperature. After staining with fluorophore-conjugated secondary antibodies, nuclei were stained with NucBlue^®^, per the manufacturer’s instructions (Life Technologies, Grand Island, NY, USA). Cover slips were mounted using Vectasheld^®^ (Vector Labs, Burlingame, CA, USA), allowed to harden overnight at 4 °C and then sealed. Images were acquired on a Zeiss LSM700 confocal microscope (Carl Zeiss, Jena, Germany) and analyzed with ImageJ software (NIH, Bethesda, MD, USA). Microarrays analyzed via immunofluorescence included tumor samples (1 mm × 5 μm) of melanomas ranging from benign nevi, RGP, VGP and distant metastases (lymph nodes, bone and spleen).

### 4.5. Overexpression Assays

MET cells were transfected with Flag-STAT1 plasmid (Addgene, Cambridge, MA, USA) with Avalanche^®^-Omni (EZ Biosystems, College Park, MD, USA), per the manufacturer’s instructions. After 24 h of incubation, cells were stimulated with 50 U/mL of IFN-γ for 48 h. Cells were harvested and analyzed as described in Section 4.2 and Section 4.3.

### 4.6. Statistical Analysis

All Western blots were quantified with Un-Scan-It Gel™ analysis software (Silk Scientific, Orem, UT, USA). Unpaired Student’s *t*-tests were used to determine significance (NS *p* > 0.05, * *p* < 0.05, ** *p* < 0.005, *** *p* < 0.001, **** *p* < 0.0001) between treated and untreated samples.

## 5. Conclusions

Despite many studies reporting the downregulation of major histocompatibility molecules in melanoma, the mechanisms underlying the silencing have not been elucidated. Our study is the first to investigate the mechanisms of MHC silencing during disease progression from the radial growth phase (RGP), vertical growth phase (VGP) and metastatic melanomas (MET). Our data will impact personalized medicine by expanding the database of therapeutic targets for the diagnosis and treatment of metastatic melanoma.

## References

[1] Clark W.H., Ainsworth A.M., Bernardino E.A., Yang C.H., Mihm C.M., Reed R.J. (1975). The developmental biology of primary human malignant melanomas. Semin. Oncol..

[2] Cichorek M., Wachulska M., Stasiewicz A., Tyminska A. (2013). Skin melanocytes: Biology and development. Postepy Dermatol. Alergol..

[3] Crutcher W.A. (1987). Dysplastic Nevi—Markers and precursors of malignant melanoma. West. J. Med..

[4] Ciarletta P., Foret L., Ben Amar M. (2011). The radial growth phase of malignant melanoma: Multi-phase modelling, numerical simulations and linear stability analysis. J. R. Soc. Interface.

[5] Herlyn M., Thurin J., Balaban G., Bennicelli J.L., Herlyn D., Elder D.E., Bondi E., Guerry D., Nowell P., Clark W.H. (1985). Characteristics of cultured human melanocytes isolated from different stages of tumor progression. Cancer Res..

[6] Laga A.C., Murphy G.F. (2010). Cellular heterogeneity in vertical growth phase melanoma. Arch. Pathol. Lab. Med..

[7] Rak J.W., Hegmann E.J., Lu C., Kerbel R.S. (1994). Progressive loss of sensitivity to endothelium-derived growth inhibitors expressed by human melanoma cells during disease progression. J. Cell. Physiol..

[8] Liotta L.A., Guirguis R., Stracke M. (1987). Biology of melanoma invasion and metastasis. Pigment Cell Res..

[9] Mlecnik B., Bindea G., Pages F., Galon J. (2011). Tumor immunosurveillance in human cancers. Cancer Metastasis Rev..

[10] Srivastava N., McDermott D. (2014). Update on benefit of immunotherapy and targeted therapy in melanoma: The changing landscape. Cancer Manag. Res..

[11] Gajewski T.F. (2006). Identifying and overcoming immune resistance mechanisms in the melanoma tumor microenvironment. Clin. Cancer Res..

[12] Braciale T.J., Morrison L.A., Sweetser M.T., Sambrook J., Gething M.J., Braciale V.L. (1987). Antigen presentation pathways to class I and class II MHC-restricted T lymphocytes. Immunol. Rev..

[13] Gerloni M., Zanetti M. (2005). CD4 T cells in tumor immunity. Springer Semin. Immunopathol..

[14] Garrido F., Cabrera T., Aptsiauri N. (2010). “Hard” and “soft” lesions underlying the HLA class I alterations in cancer cells: Implications for immunotherapy. Int. J. Cancer.

[15] Matsushita H., Vesely M.D., Koboldt D.C., Rickert C.G., Uppaluri R., Magrini V.J., Arthur C.D., White J.M., Chen Y.S., Shea L.K. (2012). Cancer exome analysis reveals a T-cell-dependent mechanism of cancer immunoediting. Nature.

[16] Nishimura T., Iwakabe K., Sekimoto M., Ohmi Y., Yahata T., Nakui M., Sato T., Habu S., Tashiro H., Sato M. (1999). Distinct role of antigen-specific T helper type 1 (Th1) and Th2 cells in tumor eradication *in vivo*. J. Exp. Med..

[17] Gao F.G., Khammanivong V., Liu W.J., Leggatt G.R., Frazer I.H., Fernando G.J. (2002). Antigen-specific CD4^+^ T-cell help is required to activate a memory CD8^+^ T cell to a fully functional tumor killer cell. Cancer Res..

[18] Antony P.A., Piccirillo C.A., Akpinarli A., Finkelstein S.E., Speiss P.J., Surman D.R., Palmer D.C., Chan C.C., Klebanoff C.A., Overwijk W.W. (2005). CD8^+^ T cell immunity against a tumor/self-antigen is augmented by CD4^+^ T helper cells and hindered by naturally occurring T regulatory cells. J. Immunol..

[19] Janssen E.M., Lemmens E.E., Wolfe T., Christen U., von Herrath M.G., Schoenberger S.P. (2003). CD4^+^ T cells are required for secondary expansion and memory in CD8^+^ T lymphocytes. Nature.

[20] Ostrand-Rosenberg S., Thakur A., Clements V. (1990). Rejection of mouse sarcoma cells after transfection of MHC class II genes. J. Immunol..

[21] Ostrand-Rosenberg S., Roby C.A., Clements V.K. (1991). Abrogation of tumorigenicity by MHC class II antigen expression requires the cytoplasmic domain of the class II molecule. J. Immunol..

[22] Muranski P., Boni A., Antony P.A., Cassard L., Irvine K.R., Kaiser A., Paulos C.M., Palmer D.C., Touloukian C.E., Ptak K. (2008). Tumor-specific Th17-polarized cells eradicate large established melanoma. Blood.

[23] Corthay A., Lundin K.U., Lorvik K.B., Hofgaard P.O., Bogen B. (2009). Secretion of tumor-specific antigen by myeloma cells is required for cancer immunosurveillance by CD4^+^ T cells. Cancer Res..

[24] Xie Y., Akpinarli A., Maris C., Hipkiss E.L., Lane M., Kwon E.K., Muranski P., Restifo N.P., Antony P.A. (2010). Naive tumor-specific CD4^+^ T cells differentiated *in vivo* eradicate established melanoma. J. Exp. Med..

[25] Haabeth O.A., Lorvik K.B., Hammarstrom C., Donaldson I.M., Haraldsen G., Bogen B., Corthay A. (2011). Inflammation driven by tumour-specific Th1 cells protects against B-cell cancer. Nat. Commun..

[26] Corthay A., Skovseth D.K., Lundin K.U., Rosjo E., Omholt H., Hofgaard P.O., Haraldsen G., Bogen B. (2005). Primary antitumor immune response mediated by CD4^+^ T cells. Immunity.

[27] Quezada S.A., Simpson T.R., Peggs K.S., Merghoub T., Vider J., Fan X., Blasberg R., Yagita H., Muranski P., Antony P.A. (2010). Tumor-reactive CD4^+^ T cells develop cytotoxic activity and eradicate large established melanoma after transfer into lymphopenic hosts. J. Exp. Med..

[28] Perez-Diez A., Joncker N.T., Choi K., Chan W.F., Anderson C.C., Lantz O., Matzinger P. (2007). CD4 cells can be more efficient at tumor rejection than CD8 cells. Blood.

[29] Fujiwara H., Fukuzawa M., Yoshioka T., Nakajima H., Hamaoka T. (1984). The role of tumor-specific Lyt-1+2- T cells in eradicating tumor cells *in vivo*. I. Lyt-1+2- T cells do not necessarily require recruitment of host’s cytotoxic T cell precursors for implementation of *in vivo* immunity. J. Immunol..

[30] Greenberg P.D., Kern D.E., Cheever M.A. (1985). Therapy of disseminated murine leukemia with cyclophosphamide and immune Lyt-1+2-T cells. Tumor eradication does not require participation of cytotoxic T cells. J. Exp. Med..

[31] Lauritzsen G.F., Weiss S., Dembic Z., Bogen B. (1994). Naive idiotype-specific CD4^+^ T cells and immunosurveillance of B-cell tumors. Proc. Natl. Acad. Sci. USA.

[32] Rudensky A., Preston-Hurlburt P., Hong S.C., Barlow A., Janeway C.A. (1991). Sequence analysis of peptides bound to MHC class II molecules. Nature.

[33] Sartoris S., Scupoli M.T., Scarpellino L., Paiola F., Jotterand-Bellomo M., Tridente G., Accolla R.S. (1990). Inducible and constitutive MHC class II gene expression. Distinct tissue-specific genetic controls. J. Immunol..

[34] Billiau A., Matthys P. (2009). Interferon-γ: A historical perspective. Cytokine Growth Factor Rev..

[35] Schroder K., Hertzog P.J., Ravasi T., Hume D.A. (2004). Interferon-γ: An overview of signals, mechanisms and functions. J. Leukocyte Biol..

[36] Igarashi K., Garotta G., Ozmen L., Ziemiecki A., Wilks A.F., Harpur A.G., Larner A.C., Finbloom D.S. (1994). Interferon-γ induces tyrosine phosphorylation of interferon-γ receptor and regulated association of protein tyrosine kinases, JAK1 and JAK2, with its receptor. J. Biol. Chem..

[37] Sakatsume M., Igarashi K., Winestock K.D., Garotta G., Larner A.C., Finbloom D.S. (1995). The JAK kinases differentially associate with the α and β (accessory factor) chains of the interferon γ receptor to form a functional receptor unit capable of activating STAT transcription factors. J. Biol. Chem..

[38] Shuai K., Horvath C.M., Huang L.H., Qureshi S.A., Cowburn D., Darnell J.E. (1994). Interferon activation of the transcription factor STAT91 involves dimerization through SH2-phosphotyrosyl peptide interactions. Cell.

[39] Nguyen H., Hiscott J., Pitha P.M. (1997). The growing family of interferon regulatory factors. Cytokine Growth Factor Rev..

[40] Masternak K., Muhlethaler-Mottet A., Villard J., Zufferey M., Steimle V., Reith W. (2000). CIITA is a transcriptional coactivator that is recruited to MHC class II promoters by multiple synergistic interactions with an enhanceosome complex. Genes Dev..

[41] Zika E., Greer S.F., Zhu X.S., Ting J.P. (2003). Histone deacetylase 1/mSin3a disrupts γ interferon-induced CIITA function and major histocompatibility complex class II enhanceosome formation. Mol. Cell. Biol..

[42] Spilianakis C., Kretsovali A., Agalioti T., Makatounakis T., Thanos D., Papamatheakis J. (2003). CIITA regulates transcription onset viaSer5-phosphorylation of RNA Pol II. EMBO J..

[43] Degenhardt Y., Huang J., Greshock J., Horiates G., Nathanson K., Yang X., Herlyn M., Weber B. (2010). Distinct *MHC* gene expression patterns during progression of melanoma. Genes Chromosom. Cancer.

[44] Groth A., Kloss S., von Strandmann E.P., Koehl U., Koch J. (2011). Mechanisms of tumor and viral immune escape from natural killer cell-mediated surveillance. J. Innate Immun..

[45] Enderling H., Hlatky L., Hahnfeldt P. (2012). Immunoediting: Evidence of the multifaceted role of the immune system in self-metastatic tumor growth. Theor. Biol. Med. Model..

[46] Boss J.M. (1997). Regulation of transcription of MHC class II genes. Curr. Opin. Immunol..

[47] De Paus R.A., Kilic S.S., van Dissel J.T., van de Vosse E. (2011). Effect of amino acid substitutions in the human IFN-γ2 on IFN-γ responsiveness. Genes Immun..

[48] Gros E., Petzold S., Maintz L., Bieber T., Novak N. (2011). Reduced IFN-γ receptor expression and attenuated IFN-γ response by dendritic cells in patients with atopic dermatitis. J. Allergy Clin. Immunol..

[49] Srisatjaluk R., Kotwal G.J., Hunt L.A., Justus D.E. (2002). Modulation of γ interferon-induced major histocompatibility complex class II gene expression by porphyromonas gingivalis membrane vesicles. Infect. Immun..

[50] Morris A.C., Beresford G.W., Mooney M.R., Boss J.M. (2002). Kinetics of a γ interferon response: Expression and assembly of CIITA promoter IV and inhibition by methylation. Mol. Cell. Biol..

[51] Truax A.D., Thakkar M., Greer S.F. (2012). Dysregulated recruitment of the histone methyltransferase EZH2 to the class II transactivator (CIITA) promoter IV in breast cancer cells. PLoS One.

[52] Reinsbach S., Nazarov P.V., Philippidou D., Schmitt M., Wienecke-Baldacchino A., Muller A., Vallar L., Behrmann I., Kreis S. (2012). Dynamic regulation of microRNA expression following interferon-γ-induced gene transcription. RNA Biol..

[53] Rodriguez T., Mendez R., Del Campo A., Jimenez P., Aptsiauri N., Garrido F., Ruiz-Cabello F. (2007). Distinct mechanisms of loss of IFN-γ mediated HLA class I inducibility in two melanoma cell lines. BMC Cancer.

[54] Serrano A., Tanzarella S., Lionello I., Mendez R., Traversari C., Ruiz-Cabello F., Garrido F. (2001). Rexpression of HLA class I antigens and restoration of antigen-specific CTL response in melanoma cells following 5-aza-2'-deoxycytidine treatment. Int. J. Cancer.

[55] Respa A., Bukur J., Ferrone S., Pawelec G., Zhao Y., Wang E., Marincola F.M., Seliger B. (2011). Association of IFN-γ signal transduction defects with impaired HLA class I antigen processing in melanoma cell lines. Clin. Cancer Res..

[56] Radosevich M., Song Z., Gorga J.C., Ksander B., Ono S.J. (2004). Epigenetic silencing of the *CIITA* gene and posttranscriptional regulation of class II *MHC* genes in ocular melanoma cells. Investig. Ophthalmol. Visual Sci..

[57] Amalraj J., Cutler S.J., Ghazawi I., Boyle G.M., Ralph S.J. (2013). Rest negatively and ISGF3 positively regulate the human *STAT1* gene in melanoma. Mol. Cancer Ther..

[58] Schoenherr C.J., Paquette A.J., Anderson D.J. (1996). Identification of potential target genes for the neuron-restrictive silencer factor. Proc. Natl. Acad. Sci. USA.

[59] Cemazar M., Golzio M., Sersa G., Hojman P., Kranjc S., Mesojednik S., Rols M.P., Teissie J. (2009). Control by pulse parameters of DNA electrotransfer into solid tumors in mice. Gene Ther..

[60] Lin F., Shen X., Kichaev G., Mendoza J.M., Yang M., Armendi P., Yan J., Kobinger G.P., Bello A., Khan A.S. (2012). Optimization of electroporation-enhanced intradermal delivery of DNA vaccine using a minimally invasive surface device. Hum. Gene Ther. Methods.

[61] Rejman J., Tavernier G., Bavarsad N., Demeester J., de Smedt S.C. (2010). mRNA transfection of cervical carcinoma and mesenchymal stem cells mediated by cationic carriers. J. Control. Release.

[62] Tuxen I.V., Jonson L., Santoni-Rugiu E., Hasselby J.P., Nielsen F.C., Lassen U. (2014). Personalized oncology: Genomic screening in phase 1. APMIS.

[63] Robertson D., Savage K., Reis-Filho J.S., Isacke C.M. (2008). Multiple immunofluorescence labelling of formalin-fixed paraffin-embedded (FFPE) tissue. BMC Cell Biol..

